# A portal into hemodynamics: utility of portal vein ultrasound in predicting variceal etiology of upper gastrointestinal hemorrhage in the emergency department

**DOI:** 10.1007/s11739-025-04229-2

**Published:** 2025-12-23

**Authors:** Siddhi Naik, Prithvishree Ravindra, Souvik Chaudhuri, P. S. Priya, Sai Deepak Alli, Shiran Shetty

**Affiliations:** 1 Department of Emergency Medicine, Kasturba Medical College, Manipal, Manipal Academy of Higher Education, Manipal, Karnataka 576104 India; 2 Department of Critical Care Medicine, Kasturba Medical College, Manipal, Manipal Academy of Higher Education, Manipal, Karnataka 576104 India; 3 Department of Radiodiagnosis and Imaging, Kasturba Medical College, Manipal, Manipal Academy of Higher Education, Manipal, Karnataka 576104 India; 4 Department of Gastroenterology, Kasturba Medical College, Manipal, Manipal Academy of Higher Education, Manipal, Karnataka 576104 India

**Keywords:** Gastrointestinal hemorrhage, Hematemesis, Diagnostic ultrasound, Doppler ultrasound, Emergency department

## Abstract

Rapid differentiation between variceal and non-variceal upper gastrointestinal hemorrhage (UGIH) in the emergency department (ED) is critical for guiding vasoactive therapy, antibiotic use, and timely endoscopy. However, endoscopy is often delayed, deferring these key management decisions and potentially impacting patient outcomes. We aimed to study the utility of portal vein ultrasound by the emergency physician for the prediction of variceal etiology in UGIH. We also studied the diagnostic accuracy of laboratory markers and fibrosis scores for differentiating between variceal and non-variceal hemorrhage. In our prospective observational study, we enrolled patients with UGIH presenting to the ED. Portal vein indices were studied using bedside point-of-care ultrasound (POCUS) by the emergency physician. Laboratory results were noted, and fibrosis scores were calculated. The upper gastrointestinal endoscopy findings were noted for the presence and grade of varices. Out of 181 patients included in the study, 106 patients (58.56%) had varices. Multivariable logistic regression analysis identified spleen diameter, platelet count, PT, ascites, and portal vein flow velocity (PVFV) were significant predictors of variceal etiology. SCoPE score (Splenomegaly, Coagulopathy, PVFV Evaluation) which is a composite that includes spleen diameter, PT, and PVFV is a useful ED-based tool for early identification of variceal bleed (AUC = 0.843, OR = 1.633). Fibrosis scores (AAR, APRI, Fib-4, King, Lok) were also useful in identifying variceal hemorrhage (*p* < 0.001). Integrating POCUS parameters and laboratory markers into clinical decision algorithms may enhance the ability of emergency physicians to predict variceal etiology of UGIH. Novel ED-based composite SCoPE score can help the physicians predict varices and enable them to tailor the management plan and optimize resuscitation strategies.

**Supplementary Information:**

The online version contains supplementary material available at https://doi.org/10.1007/s11739-025-04229-2.

## Introduction

Upper gastrointestinal hemorrhage (UGIH) is a life-threatening emergency and warrants prompt diagnosis and management. The emergency physician often must make the treatment plan at the bedside, without knowing the etiological cause. Recent trends indicate that variceal causes of UGIH are more prevalent than non-variceal causes, such as peptic ulcer disease [1–3]. Variceal UGIH is a critical complication of portal hypertension that primarily occurs in patients with chronic liver disease (CLD) and necessitates the administration of vasoactive agents for splanchnic vasoconstriction. Additionally, the guidelines recommend administration of antimicrobial prophylaxis and timely endoscopic treatment within 12 h in a variceal hemorrhage [4]. These treatment implications highlight the need for emergency physicians to differentiate between variceal and non-variceal causes of UGIH.

While upper GI endoscopy (UGIE) remains the gold standard for diagnosing varices, immediate endoscopy may not be available in resource-limited settings, and visualization may be challenging in actively bleeding patients. It often takes up to two days for patients to reach the endoscopy suite after admission [5]. Therefore, there is a need to explore non-invasive methods for predicting varices that are quick, easily reproducible, and can be performed in the emergency department (ED).

Previously, non-invasive methods have been developed to predict varices in patients with CLD in an outpatient setting. Ultrasonography performed by radiologists has been used to predict portal hypertension and varices in cirrhosis [6, 7]. Additionally, hematological and biochemical parameters, along with composite fibrosis scores, have been used to detect advanced liver diseases, which have been identified as surrogate markers to predict varices [8, 9]. However, there is a lacuna of studies on Emergency Department (ED)-based predictors of varices in patients with active UGIH, where the diagnosis of CLD may be uncertain.

Point-of-care ultrasound (POCUS) has emerged as an essential tool in ED physicians’ armamentarium, allowing for rapid, real-time diagnostic and management decisions at the bedside. The applications of portal vein ultrasound in the ED remain underexplored. Portal vein hemodynamics measured using portal vein diameter (PVD), blood flow patterns, portal vein flow velocity (PVFV), and spleen diameter can give us crucial insights into the pathophysiology associated with UGIH.

Our primary objective was to investigate the diagnostic utility of the portal vein ultrasound in patients with active UGIH in the ED to predict variceal etiology. Our secondary objective was to evaluate the utility of laboratory parameters and fibrosis scores to identify varices in UGIH. Non-invasive diagnostic modalities, such as POCUS and fibrosis scores, may help identify patients with variceal hemorrhage early and guide optimal resuscitation strategies in the ED.

## Methods

### Study design and period

We conducted a prospective observational study in a tertiary care academic hospital. This study was conducted over 24 months (from June 2023 to May 2025) and approved by Kasturba Medical College and Kasturba Hospital Institutional Ethics Committee (IEC2: 134/2023). It is also registered with clinical trial registry of India CTRI/2023/05/053156.

### Study population

Patients aged > 18 years presenting to the ED with UGIH were included after their written informed consent. Patients with portal vein thrombosis or cavernoma were excluded. Patients who did not undergo UGIE were also excluded from the study.

### Sample size

As there is lack of ED-based studies assessing the portal vein ultrasound parameters in UGIH patients to predict varices, to calculate sample size, we used this study by Wasif Khan et al. [10] done in CLD patients. As per the primary objective, considering that the sensitivity of PVFV to predict varices is about 90% [10], with prevalence of variceal bleed among UGI bleeds presenting to emergency being about 30% [11], a precision of 8%, and expected drop out of 5%, the final sample size required was 181.

### Examination selection

**Fig. 1:**
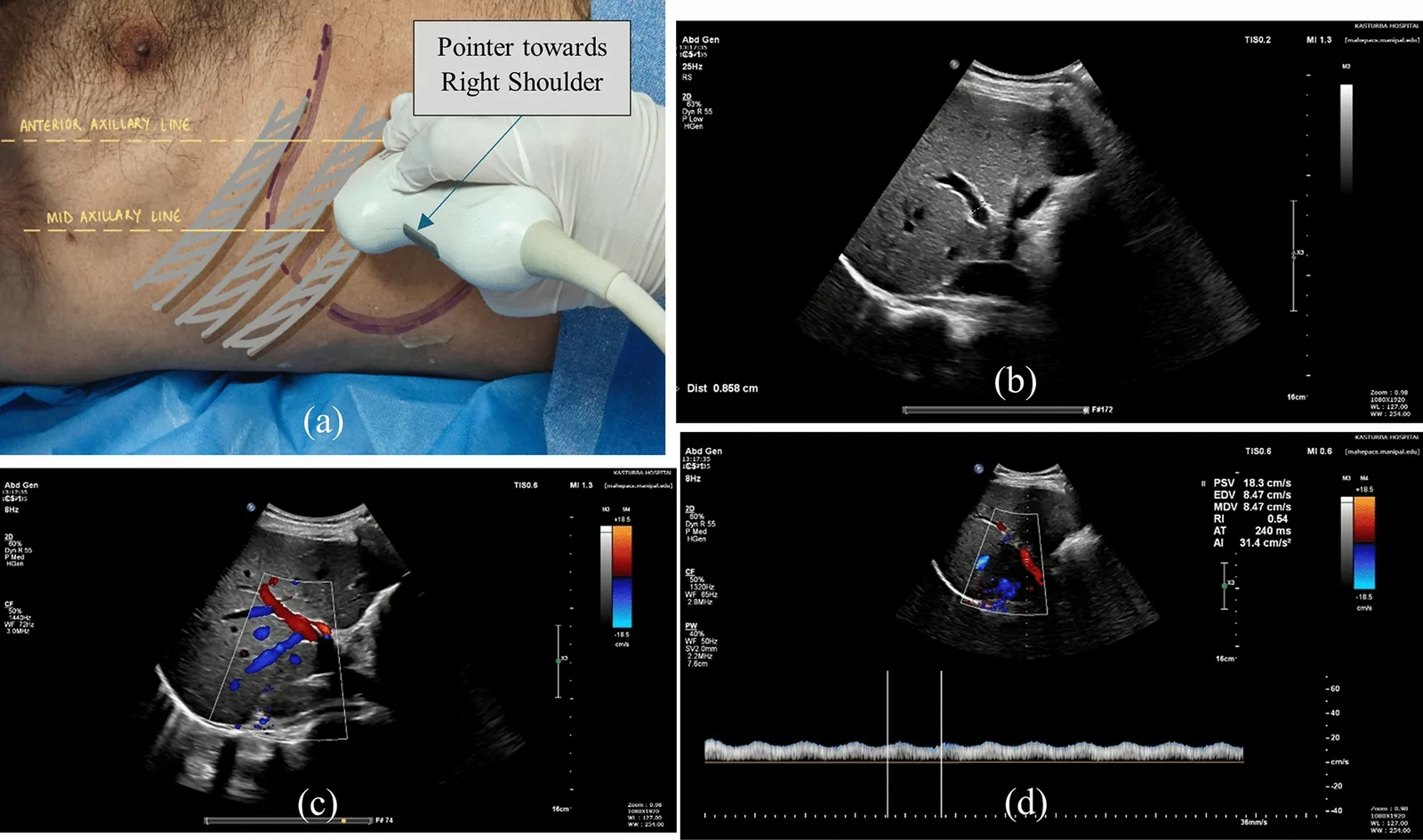
**a** Anatomical landmarks for ultrasound probe placement. **b** Portal vein diameter. **c** Portal vein blood flow-color Doppler. **d** Portal vein flow velocity

POCUS was done bedside in the ED using Philips CX-50 portable ultrasound and the GE Versana Active™ Ultrasound using the curved array probe with a broadband frequency range of 5 MHz and 3–4 MHz, respectively. A transabdominal ultrasound was done in the supine or semi-recumbent position. The probe was positioned in the sub-xiphoid area to identify the hepatic veins and their connection to the inferior vena cava (IVC). A sub-segmental view of the liver was attained by sliding the probe to the right intercostal space and orienting it obliquely to visualize the proximal extrahepatic porta hepatis (Fig. 1).

**Fig. 2:**
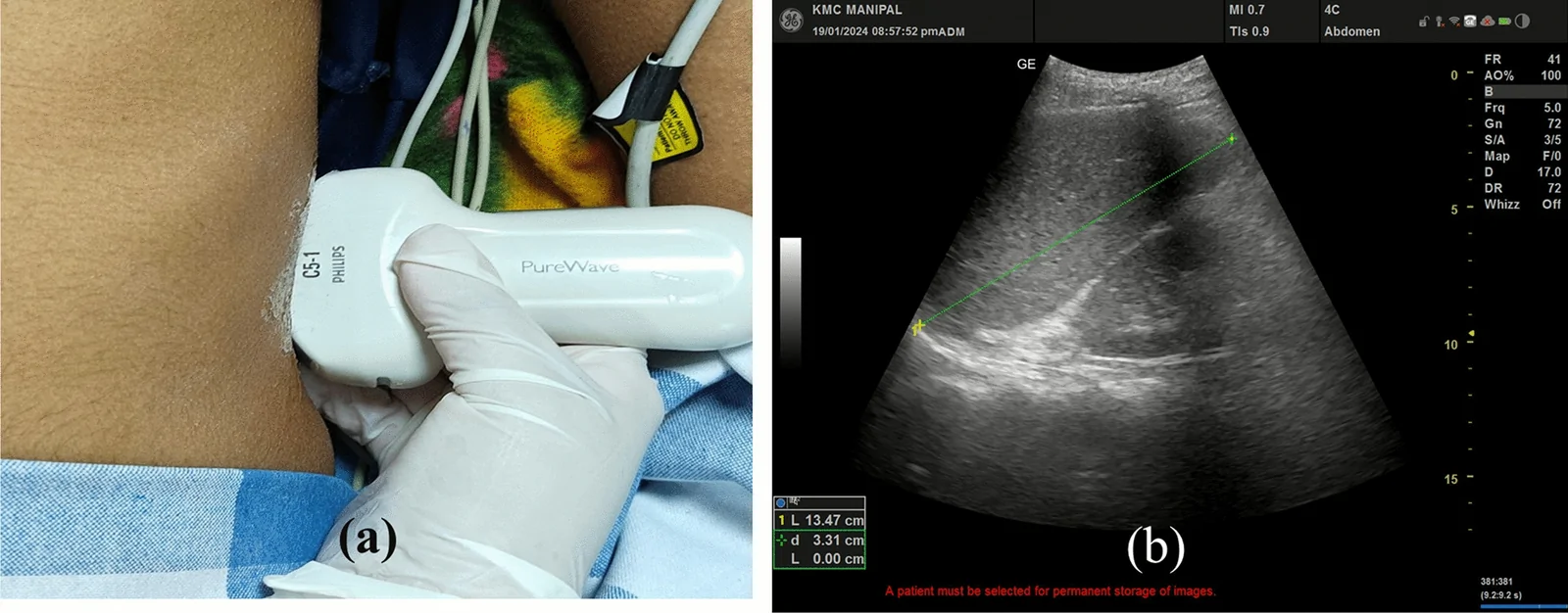
**a** Probe position in left intercostal space. **b** Measurement of spleen diameter and assessment of free fluid

Portal vein diameter (PVD) was measured in deep expiration before its bifurcation as the mean of three values of the intraluminal distance. Portal vein flow velocity (PVFV) was measured in the mid-portion of the portal vein with a Doppler beam, and the long axis of the portal vein was maintained at less than 60°. The probe was then rotated 90° to the subcostal oblique position to measure the portal vein's cross-sectional area (CSA) at the porta hepatis. A color Doppler ultrasound of the portal vein assessed its patency and blood flow direction. Flow appears blue when moving away from the liver (hepato-fugal) and red when moving toward the liver (hepato-petal). The portal congestion index (PCI) was calculated as a ratio of portal vein CSA and PVFV. Spleen was then measured in the left 8th–10th intercostal space, and the maximum length was taken in the mid-longitudinal view (Fig. 2). Ascites was detected with a focused free fluid assessment in the hepato-renal, spleno-renal, and suprapubic views.

### Image acquisition and review

The images were acquired by 2 investigators (an emergency physician and an emergency medicine resident under the supervision of the physician). 2 investigators underwent training in portal ultrasound with a radiologist. To gain competence, they completed 25 portal vein ultrasounds under the supervision of the radiologist, prior to starting enrollment for the study. At the time of scan, the investigator performing the POCUS was blinded to comorbidities, including CLD, to minimize observer bias during image acquisition and interpretation.

### Data collection and variables

Patient demographic details and clinical presentation were collected in a structured proforma. Laboratory investigation results, including hemoglobin, hematocrit, platelet count, liver function tests, and coagulation parameters, were recorded. Fibrosis scores (AAR, APRI, FIB-4, King Score, and Lok Index) were calculated using standard published formulae (Table 1). [9] These composite scores, derived from routine laboratory parameters, such as AST, ALT, platelet count, INR, were evaluated as predictors of varices in UGIH.

**Table 1:** Formulae used for the calculation of fibrosis scores

Fibrosis scores	Calculation
AAR	AST/ALT
APRI	[(AST/ULN) × 100]/PLT
Fib-4	(Age × AST)/(PLT × √ALT)
King	(Age × AST × INR)/PLT
Lok index	LogOddsLok = [1.26 × (AST/ALT)] + (5.27 × INR) − (0.0089 × PLT) − 5.56 LokIndex = e^(LogOddsLok)^/(1 + e^(LogOddsLok)^)

*APRI* AST-to-platelet ratio index, *AST* aspartate transaminase, *PLT* platelet count, *ALT* alanine transaminase, *INR* international normalized ratio

As part of the standard of care, the patients underwent UGIE with Olympus Evis Exera III CV-190 Endoscope. The results were noted, including the presence of varices and the grade of varices.

### Data analysis

Statistical analysis for this study was performed using SPSS version 30.0. Measurement data were summarized as mean ± standard deviation for normally distributed data and median with IQR for non-normally distributed data. We used *t* tests for normally distributed data and Mann–Whitney *U* tests for non-normally distributed data.

Our investigation focused on comparing POCUS and UGIE findings, particularly assessing the presence of varices. Univariate and multivariate logistic regression analyses were conducted to explore the relationship between POCUS, laboratory parameters, and varices on UGIE. Additionally, univariate analysis on fibrosis scores was also done. Receiver Operating Characteristic (ROC) analysis was used to establish cut-off values for the identified significant parameters, facilitating their potential use as diagnostic tools. We also assessed the diagnostic accuracy (sensitivity, specificity, positive predictive value, negative predictive value, and likelihood ratio) of the derived cut-offs to predict varices.

## Results

### Demographics and clinical characteristics

During the study period, 210 patients presented to the ED with UGIH, of which 181 were eligible for inclusion (Supplementary Fig. 1). The mean age of the patients was 55 years (± 14.741 SD) with a male preponderance (88%). Clinical characteristics and discharge outcomes of study cohort are summarized in Table 2.

**Table 2:** Clinical characteristics and discharge outcomes of the patient cohort (*N* = 181)

Patient characteristics	Number of patients	%
*Co-morbidities*		
Total CLD	122	67.4%
Previously diagnosed CLD	90	49.72
Type 2 diabetes mellitus	46	25.41
Arterial hypertension	38	20.99
Ischemic heart disease	10	5.52
*Others*		
CVA	9	4.97
CKD	4	2.21
Adenocarcinoma pancreas	1	0.55
ITP	1	0.55
*Risk factors*		
Alcohol consumption	90	49.72
Past GI hemorrhages	81	44.75
Anti-platelets use	46	25.41
Peptic ulcer disease	15	8.29
Recent NSAIDs use	7	3.87
Anti-coagulant use	1	0.55
*Clinical presentations*		
Hematemesis and melena	114	62.98
Hematemesis alone	37	20.44
Melena alone	26	14.36
Hematemesis and hematochezia	10	5.52
Hematochezia alone	2	1.10
Ascites	45	24.86
*Patient outcomes*		
Discharge	171	94.48
Discharged against medical advice	7	3.86
Death	3	1.65

*CVA* cerebrovascular accident, *CKD* chronic kidney disease, *ITP* immune thrombocytopenic purpura, *GI* gastrointestinal; *NSAIDs* non-steroidal anti-inflammatory drugs

Among the study population (*N* = 181), 122 patients had CLD. Of these, 90 had a prior diagnosis of CLD (80 variceal UGIH, 10 non-variceal UGIH), and 32 were newly diagnosed CLD during admission (25 variceal UGIH, 7 non-variceal UGIH). One patient with variceal bleeding had non-cirrhotic portal hypertension secondary to myelofibrosis.

In our cohort, two patients died due to hemorrhagic shock and one due to sepsis. This does not reflect the true UGIH mortality in our setting as the patients who died prior to UGIE were excluded by study criteria. Additionally, seven critically ill patients were discharged against medical advice, and their outcomes remain unknown.

**Fig. 3:**
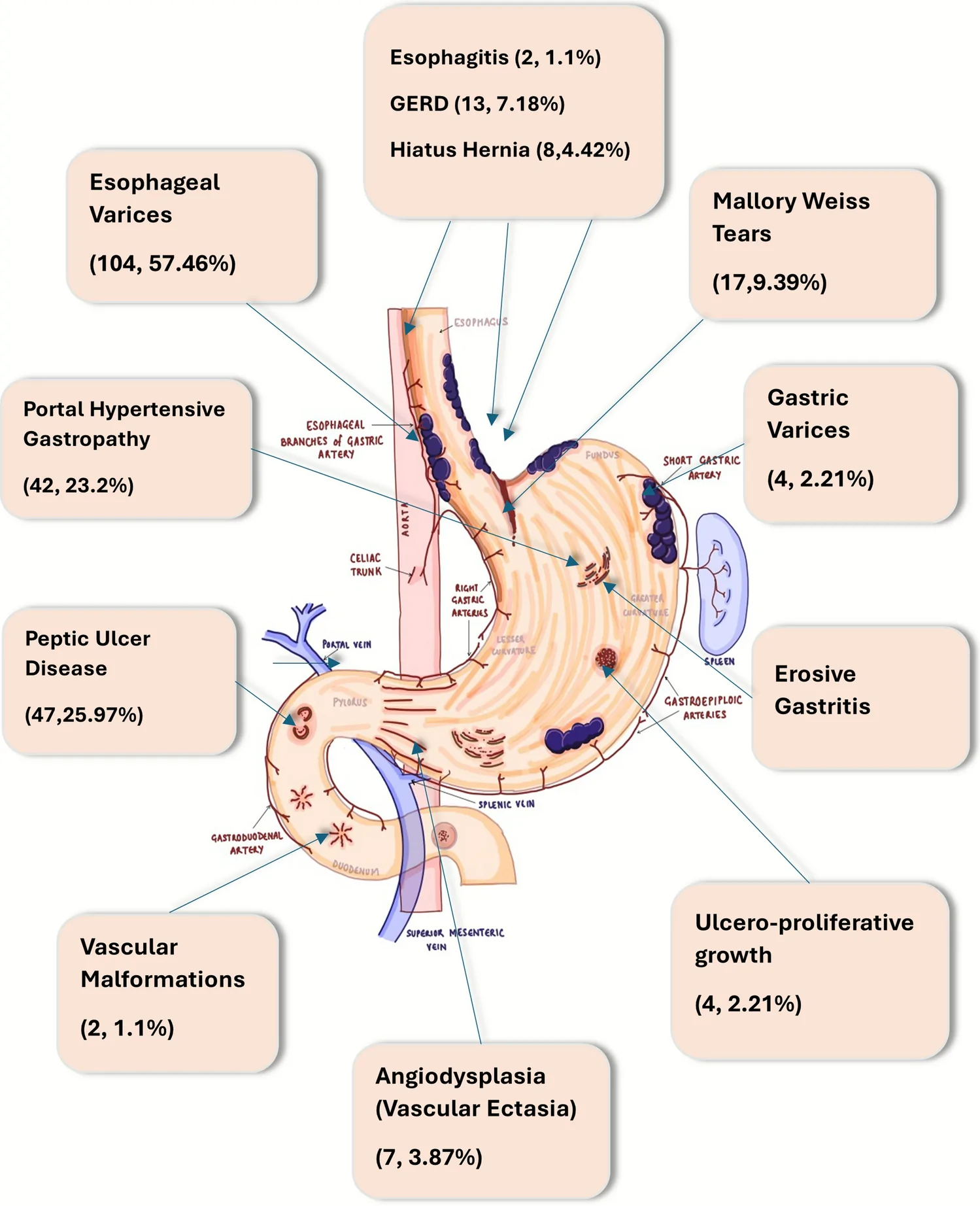
Upper GI endoscopy findings in patients with upper GI hemorrhage (*N* = 181); The frequency of the pathologies found in upper GI endoscopy is represented as (*n*, %)

UGIE findings of our patient population, along with their frequency, are diagrammatically represented in Fig. 3. The most common etiology of UGIH was varices (106, 58.56%), followed by peptic ulcer disease (47, 25.97%). Among the 106 patients with variceal bleeding, 102 had only esophageal varices, 2 had only gastric varices, and 2 patients had both. Most of the patients with variceal hemorrhage had large varices (80, 75%).

A prior history of CLD was found to be significantly associated with the presence of varices on UGIE. Among the 90 patients with known CLD, 80 (88.9%) had varices on UGIE, whereas only 26 of the 91 patients without a previous CLD diagnosis (28.6%) had varices. This association was statistically significant (*χ*^2^ *p* < 0.001), with a Phi/Cramer’s *V* value of 0.612 indicating a strong effect size.

### POCUS and laboratory predictors of varices in UGIH

Portal vein indices measured at bedside using POCUS performed by an emergency physician were able to predict varices in UGIE. On univariate analysis, PVD, spleen diameter, PVFV, portal vein CSA, PCI, presence of ascites, platelet count/spleen diameter ratio (PC/SD) were statistically significant predictors of variceal etiology, as shown in Table 3. Univariate analysis of laboratory parameters suggested that hemoglobin, hematocrit, platelet count, liver function tests, and coagulation profile could predict variceal etiology, as represented in Table 4.

**Table 3:** Comparison of POCUS parameters between patients with varices and patients without varices on UGIE

Parameter	Varices absent (*n* = 75)	Varices present (*n* = 106)	*p* value
Portal vein diameter (mm)	9.03 ± 2.63	9.87 ± 2.71	0.038*
Spleen diameter (cm)	9.76 ± 2.14	12.47 ± 2.30	< 0.001*
Portal vein velocity (cm/s)	14.40 (11.00–19.00)	11.65 (9.00–14.00)	< 0.001^#^
Portal vein CSA (cm^2^)	0.63 (0.50–0.87)	0.75 (0.52–1.08)	0.046^#^
Portal vein congestion index	0.04 (0.32–0.59)	0.06 (0.04–0.09)	< 0.001^#^
Platelet (cu mm)/spleen diameter(mm)	2093 (1254–3038)	1069 (726–1552)	< 0.001^#^
Ascites present	10 (13.3%)	47 (44.3%)	< 0.001^^^
*Blood flow pattern*			
Hepatopetal	62.7%	55.7%	0.085^^^
Hepatofugal	9.3%	21.7%	
Mixed	28%	22.6%	

*Independent Student *t* test, ^#^Mann–Whitney *U* test, ^Chi-square test

**Table 4:** Comparison of laboratory markers between patients with varices and patients without varices on UGIE

S. no.	Variables	No varices (*n* =75)	Varices present (*n* = 106)	*p* value
1	Hematocrit (%)	28.19 ± 9.8	25.17 ± 7.6	0.027*
2	Hemoglobin (g/dL)	9.46 ± 3.50	8.44 ± 2.70	0.030*
3	WBC (× 10^3^/µL)	10.2 (6.82–14.30)	10 (7.1–14.0)	0.912^#^
4	Platelets (× 10^3^/µL)	203 (130–258)	135 (86.50–187.75)	< 0.001^#^
5	Creatinine (mg/dL)	1.03 (0.79–1.52)	0.99 (0.72–1.41)	0.590^#^
6	Urea (mg/dL)	46.00 (24–77)	41.00 (31–64.25)	0.422^#^
7	Total bilirubin (mg/dL)	0.77 (0.40–1.25)	2.08 (1.07–5.53)	< 0.001^#^
8	Direct bilirubin (mg/dL)	0.38 (0.15–0.72)	1.20 (0.54–0.42)	< 0.001^#^
9	AST (U/L)	40 (22–73)	70 (48.25–115.75)	< 0.001^#^
10	ALT (U/L)	23.4 (14.4–40)	29 (21.65–50.35)	0.004^#^
11	ALP (U/L)	72 (60–108)	101 (81.50–132.25)	< 0.001^#^
12	Albumin (g/dL)	3.30 ± 0.79	2.84 ± 0.59	< 0.001*
13	INR	1.06 (0.98–1.25)	1.39 (1.22–1.65)	< 0.001^#^
14	PT (s)	13.05 ± 3.36	16.76 ± 5.18	< 0.001*
15	aPTT (s)	27.12 ± 10.02	30.80 ± 8.47	0.005*

*Independent Student *t*-test; ^#^Mann–Whitney *U* test

Variables with univariate significance (*p* < 0.05) were screened for multi-collinearity. Parameters with potential co-dependence—such as INR with PT, and ratio-based or derived indices including the PCI and PC/SD—were excluded to avoid redundancy and overestimation of effects. The remaining variables were entered into separate multivariable logistic regression models for laboratory and POCUS predictors to identify independent determinants of varices as presented in Tables 5 and 6. After identifying the independently significant laboratory and POCUS parameters, a combined multivariable model was constructed, in which PT (OR = 1.182), spleen diameter (OR = 1.493), and portal vein flow velocity (OR = 0.921) remained significant predictors as represented in Table 7.

**Table 5:** Multivariable logistic regression analysis of POCUS parameters for predicting varices in UGIH

Variables	*p* value	Odds Ratio	95% CI for OR	
			Lower	Upper
Portal vein diameter	0.883	0.989	0.849	1.152
Portal vein flow velocity	0.004	0.902	0.841	0.968
Spleen diameter	< 0.001	1.612	1.331	1.953
Ascites	0.030	2.698	1.100	6.619

**Table 6:** Multivariable logistic regression analysis of laboratory parameters for predicting varices in UGIH

Variables	p value	Odds Ratio	95% CI for OR	
			Lower	Upper
Hematocrit	0.202	0.967	0.918	1.018
Platelets	0.003	0.992	0.987	0.997
Total bilirubin	0.334	1.049	0.952	1.156
AST	0.879	1	0.994	1.005
ALT	0.141	0.994	0.987	1.002
ALP	0.233	1.004	0.998	1.009
Albumin	0.874	1.060	0.518	2.166
PT	0.006	1.288	1.074	1.544

**Table 7:** Multivariable logistic regression: combined model of laboratory and POCUS parameters for predicting varices

Variables	Beta coefficient	*p* value	Odds ratio	95% CI for OR	
				Lower	Upper
Portal vein flow velocity	− 0.082	0.037	0.921	0.853	0.995
Spleen diameter	0.401	< 0.001	1.493	1.230	1.813
Ascites	0.402	0.424	1.495	0.558	4.001
Platelet	− 0.004	0.088	0.996	0.990	1.001
PT	0.168	0.015	1.182	1.033	1.353

**Fig. 4:**
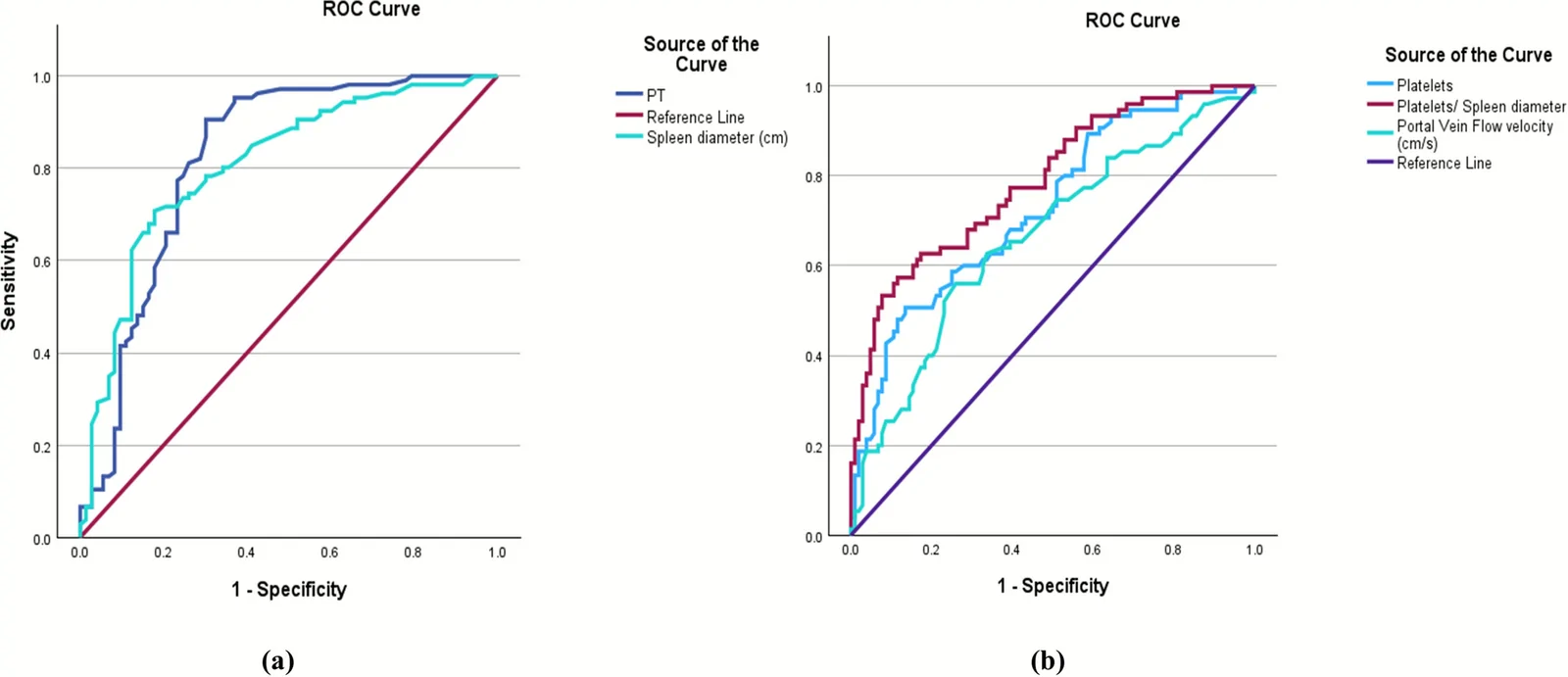
**a** ROC curve of parameters predicting the presence of varices on UGI endoscopy in UGIH. **b** ROC curve of parameters predicting the absence of varices on UGI endoscopy in UGIH

ROC analysis: The ROC curve analysis (Fig. 4) demonstrated that spleen diameter, PT, and PC/SD had the highest diagnostic performance with an area under curve (AUC) of 0.808, 0.822, and 0.789, respectively, followed by the platelet count (AUC 0.730), and PVFV (AUC 0.665). Using the ROC curve, the most appropriate cut-offs were derived, and their diagnostic accuracy is summarized in Table 8.

**Table 8:** Diagnostic accuracy of POCUS and laboratory parameters for predicting variceal etiology in UGIH

Variables	AUC	Cut-off	Sensitivity (%)	Specificity (%)	PPV (%)	NPV (%)	Diagnostic accuracy (%)	LR (+)	LR (−)
*Diagnostic accuracy for predicting the presence of varices*									
Spleen diameter	0.808*	≥ 12*	66	85	86.4	64	74.1	4.5	0.4
PT	0.822*	≥ 14.5	64.2	79.5	81.9	60.4	70.4	3.13	0.45
*Diagnostic accuracy for predicting the absence of varices*									
PVFV	0.665*	≥ 14*	56	74	60	70.3	66.3	2.12	0.6
Platelets/spleen diameter	0.789*	≥ 1741*	60	85	74	74.6	74.3	3.90	0.47
Platelet count	0.730*	≥ 155*	66.7	61.3	55	72.2	64	1.72	0.54

*p value < 0.001

### Combined predictive model: SCoPE score

Using the beta coefficients from these independently significant predictors (Table 7) and cut-offs derived from the ROC curve (Table 8), we constructed a combined predictive model and developed the **SCoPE score** (**S**plenomegaly, **Co**agulopathy, **P**VFV **E**valuation) to estimate the likelihood of varices in patients presenting with UGIH. As portal vein flow velocity had the smallest absolute beta coefficient (0.082), it was used as the reference value for proportional score assignment. Beta coefficients of the remaining variables were divided by 0.082 to derive their weighted contributions. The final scoring system is presented in Table 9.

**Table 9:** **SCoPE score** (**S**plenomegaly, **Co**agulopathy, **P**VFV **E**valuation) to estimate the likelihood of varices in patients presenting with UGIH

Parameter	Cut-off value	Points assigned
PVFV	< 14 cm/s	1
PT	≥ 14.5 s	2
Splenomegaly on POCUS	≥ 12 cm	5
**Total**		**8**

**Fig. 5:**
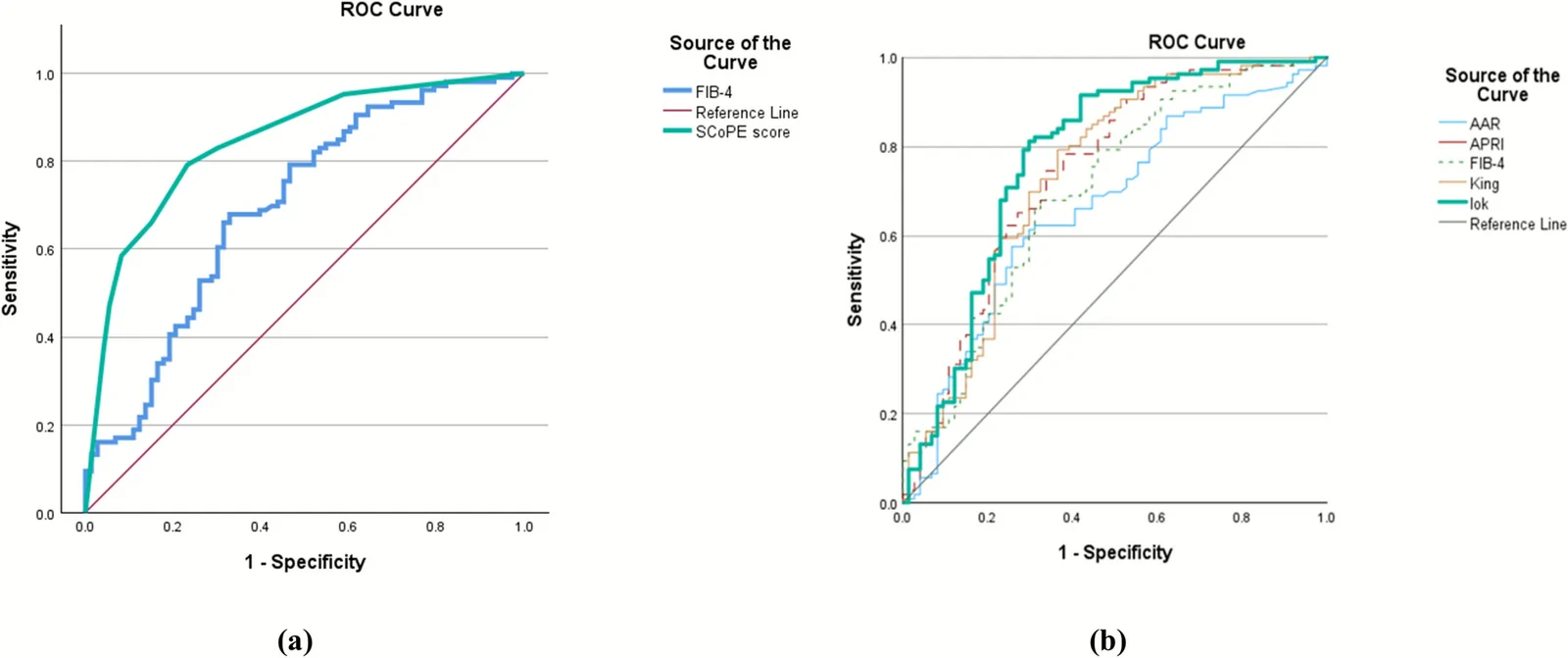
ROC curve of SCoPE score and FIB-4 for predicting the presence of varices on UGIE in UGIH patients. **b** ROC curve of fibrosis scores for predicting the presence of varices on UGI endoscopy in UGIH

This composite SCoPE score integrates POCUS and laboratory findings to provide a simple ED-based tool for predicting varices in UGIH. As demonstrated in Fig. 5 and supplementary Table 1, the score demonstrated good diagnostic performance (AUC 0.843; OR 1.633), with an optimal cut-off ≥ 3 achieving a sensitivity of 79.2%, specificity of 77%, PPV of 83.2%, and an overall diagnostic accuracy of 78.2%, underscoring its reliability for identifying patients with varices.

### Subgroup analysis of patients with CLD

Among the 181 patients, 122 had a diagnosis of CLD. In this subgroup, univariate analysis showed that spleen diameter (*p* = 0.004, AUC = 0.713) and PVFV (*p* = 0.035, AUC = 0.660) significantly predicted variceal bleeding, as highlighted in Supplementary Table 2 and Supplementary Fig. 2.

Among the study cohort, 23 patients were Child–Pugh A, 40 were Child–Pugh B, and 59 were Child–Pugh C. Portal vein flow velocity significantly predicted the presence of varices in Child–Pugh A (*p* = 0.015, AUC = 0.817) and Child–Pugh B patients (*p* = 0.002, AUC = 0.934). Spleen diameter effectively discriminated the etiology of UGIH in Child–Pugh A patients (*p* = 0.024, AUC = 0.768). These findings have been outlines in Supplementary Table 3 and Supplementary Fig. 3.

Among the newly diagnosed CLD subgroup (n = 32), PVFV was significantly associated with the presence of varices (*p* = 0.001; AUC 0.886), and PCI also showed significant discriminatory performance (*p* = 0.018; AUC 0.794). (Supplementary Table 4).

### Subgroup analysis of patients without previously diagnosed CLD

Among the study population, 90 patients had a prior diagnosis of CLD. A subgroup analysis was therefore performed on the remaining 91 participants (59 non-CLD and 32 newly diagnosed CLD) to minimize operator-related bias and evaluate diagnostic performance in the cohort most likely to remain undiagnosed and untreated for variceal bleeding until endoscopy. In this subgroup, spleen diameter, PVFV, PCI, PC/SD, presence of ascites, platelet count, PT, total bilirubin, AST, ALT, and ALP remained significant predictors of varices (Supplementary Table 5). ROC analysis demonstrated the highest discriminative ability for spleen diameter (AUC 0.819), PT (AUC 0.814), PC/SD (AUC 0.801) and PVFV (AUC 0.773) as shown in Supplementary Fig. 4. Given the limited number of variceal cases (*n* = 26), multivariable logistic regression was not feasible due to the recommended events-per-variable requirement and would risk overfitting. Therefore, subgroup findings are presented using univariate and ROC-based evaluation.

### Fibrosis scores and variceal etiology in UGIE

We analyzed the association between fibrosis scores and presence of varices in patients with active UGIH. AAR, APRI, Fib4, King, and Lok index fibrosis scores could significantly predict variceal etiology (*p* < 0.001) as demonstrated in Table 10 and had reliable AUC (Fig. 5). The most appropriate cut-offs and their diagnostic accuracies are provided in the supplementary material (Supplementary Table 1).

**Table 10:** Comparison of fibrosis scores in patients with varices and patients without varices on UGIE

Variables	No varices (*n* = 75)	Varices present (*n* = 106)	*p* value
AAR	1.62 (1.14–2.28)	2.43 (1.47–3.36)	< 0.001^#^
APRI	0.51 (0.24–1.08)	1.57 (0.80–2.86)	< 0.001^#^
Fib-4	2.51 (1.41–5.40)	5.27 (1.95–8.33)	< 0.001^#^
King	12.15 (6.21–36.25)	42.17 (22.55–77.15)	< 0.001^#^
Lok index	0.59 (0.33–0.92)	0.98 (0.88–0.99)	< 0.001^#^

^#^Mann–Whitney *U* test

### Comparison of POCUS parameters between groups of patients with no varices, small varices, and large varices

ANOVA test and Kruskal–Wallis test showed that spleen diameter, PVFV, PC/SD, and PCI were shown to correlate significantly with the grade of varices on UGIE (Table 11). Boxplot of the results is presented in Fig. 5.

**Table 11:** Difference in ultrasound parameters between groups—patients with no varices, small varices, and large varices

Variables	No varices (*n* = 75) (Group 1)		Small varices (*n* = 26) (Group 2)	Large varices (*n* = 80) (Group 3)	*p* value
Portal vein diameter		9.02 ± 2.63	10.00 ± 2.14	9.83 ± 2.88	0.113*
Spleen diameter		9.75 ± 2.14	12.17 ± 1.97	12.57 ± 2.40	< 0.001*
Portal vein flow velocity		14.4 (11–19)	12.15 (8.68–18.00)	11.50 (0.93–13.80)	< 0.001^#^
Portal vein CSA		0.63 (0.50–0.84)	0.69 (0.56–1.00)	0.79 (0.51–1.10)	0.136^#^
Portal vein congestion index		0.42 (0.32–0.59)	0.57 (0.35–0.90)	0.67 (0.42–0.99)	0.001^#^
Platelets/Spleen diameter ratio		2093 (1254–3038)	1344 (746–1695)	1028 (720–1507)	< 0.001^#^

Post hoc test *p* value is significant between group 1 and group 2, and between group 1 and group 3

*ANOVA test; ^#^Kruskal–Wallis test

### Medication and hemodynamics-related confounding

Patients on non-selective beta-blockers were not excluded to preserve the pragmatic nature of the cohort, as many cirrhotic patients presenting with UGIH are already on primary prophylaxis. To account for potential hemodynamic effects of beta-blockade on portal measurements, a subgroup analysis was performed among the 129 patients not receiving non-selective beta-blockers. In this subgroup, portal vein flow velocity (PVFV) and portal congestion index (PCI) remained significant predictors of varices (*p* < 0.001). On ROC analysis, PVFV (AUC 0.676) and PCI (AUC 0.693) demonstrated fair discriminatory ability, supporting their continued diagnostic relevance even after removing medication-related confounding (Supplementary Table 6 and Supplementary Fig. 5).

We assessed the effect of systolic blood pressure on portal hemodynamic measurements. There was no significant correlation between SBP and portal vein flow velocity as assessed by Spearman’s correlation (*p* = 0.550). Similarly, no correlation was observed between SBP and portal vein diameter on Pearson’s correlation analysis (*p* = 0.344) (Supplementary Fig. 6).

**Fig. 6:**
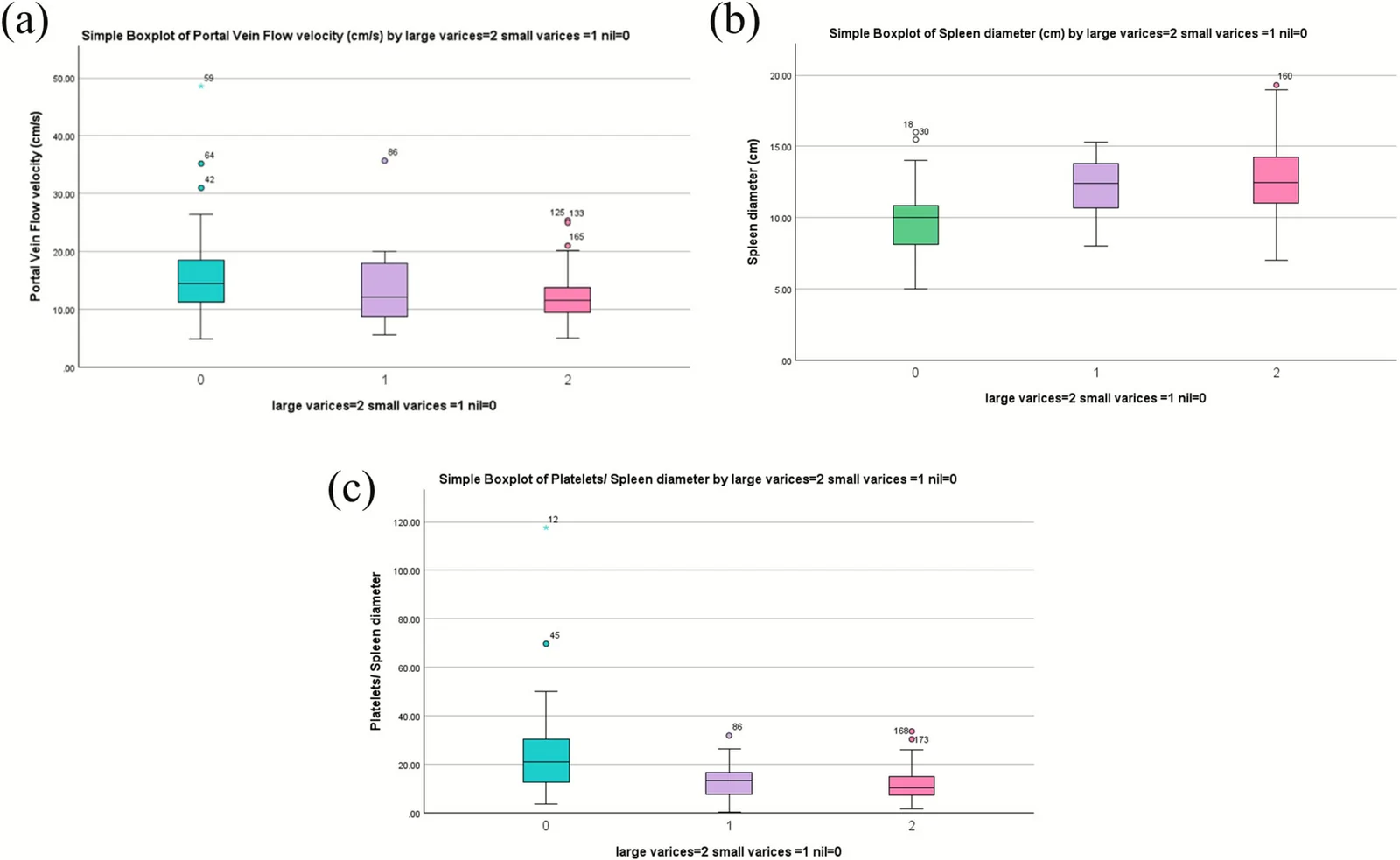
Box plot comparison of POCUS parameters and grade of varices—no varices, small varices, large varices. **a** PVFV, b spleen diameter and **c** platelet count/spleen diameter ratio

## Discussion

Through our study, we aimed to identify non-invasive predictors of variceal etiology in patients with UGIH presenting to ED. We found that spleen diameter on POCUS, PC/SD ratio, PT, and PVFV could help us rapidly identify patients with variceal hemorrhage and initiate appropriate management with vasoactive agents, antibiotics and timely endoscopy. Additionally, fibrosis scores, which are traditionally used for identifying advanced liver disease in patients, were also found to be useful in patients with UGIH to predict variceal etiology. The newly developed **SCoPE** score, integrating spleen diameter, PT, and PVFV, showed strong predictive ability for identifying variceal etiology in UGIH. With an AUC of 0.843 and an overall diagnostic accuracy of 78.2%, the score provides a simple, ED-applicable tool for early risk stratification.

In our patient population, esophageal varices (58.6%) were the most common cause of UGIH, followed by Peptic Ulcer disease (25.97%). In previous decades, UGIH was the most frequently attributed to peptic ulcer disease [1, 12]. However, with the widespread use of proton pump inhibitors and H2 receptor antagonists for treatment and prophylaxis of peptic ulcers, there has been a notable shift in the etiology of UGIH. Recent studies suggest that variceal bleeding may have become the predominant cause of UGIH [2, 3, 13], although regional variations may exist [1]. Variceal UGIH has a higher mortality than peptic ulcer disease-related bleed [2, 13], highlighting the need for prompt recognition and swift management.

Moreover, variceal hemorrhage requires tailored treatment, including vasoactive agents, such as somatostatin analogs, antibiotic administration, and early endoscopic management. However, often there is a delay in the diagnosis of the variceal etiology of UGIH due to a delay in endoscopy [5]. Recent 2024 guidance document from the AASLD emphasizes the role of non-invasive assessment in the risk stratification of portal hypertension and varices, using liver stiffness measurement and platelet count to identify clinically significant portal hypertension, in alignment with the Baveno VII consensus [4]. However, liver stiffness measurement requires patient stability, fasting, and dedicated equipment. Immediate availability of transient elastography to assess liver stiffness in the ED when a patient comes with UGIH is a challenge. POCUS-based parameters and composite scores such as SCoPE score offer a pragmatic alternative in an actively bleeding patient, especially when the diagnosis of CLD is uncertain. POCUS can be performed immediately at the bedside during active resuscitation, and hence can be a complementary addition to the algorithm, in patients with UGIH.

Among the studied POCUS parameters, spleen diameter had the highest AUC of 0.808 for predicting variceal etiology. We assessed the spleen diameter using POCUS, as previous studies have emphasized addition of POCUS to traditional physical examination improves the diagnostic accuracy of detecting splenomegaly [14]. In patients with liver cirrhosis, spleen size has been found to be a predictor of varices [8, 15–18]. Various cut-offs considered in these studies have been summarized in the supplementary table 7. As our study population was UGIH, we found that spleen length > 12 cm, which is consistent with splenomegaly cut-off, would be appropriate for identifying varices.

Our study found that thrombocytopenia supported the diagnosis of variceal etiology of UGIH. Although 25.4% of our study participants had history of antiplatelet use, this may not directly decrease the platelet count and needs to be assessed using platelet function studies. Additionally, PC/SD ratio was a useful predictor for the cause of UGIH, as well as could reliably predict presence of large varices. Among the patients with CLD, previous studies have found platelet count to be a useful hematologic marker to predict presence of varices [6, 18, 19]. Combining the platelet count with the spleen diameter considers two significant predictors to form a useful ratio, which can help in our rapid decision-making at the ED, which was also underlined in the previous studies by Nouh et al. and Cherian et al. [8, 20–22]. Although the focus of earlier studies have been on patients with CLD, we find platelet count and PC/SD ratio to be important markers in UGIH patients, which can inform treatment strategies, rendering them an essential tool in clinical practice.

Portal hypertension causes a reduction in PVFV, which was found to be a useful portal vein index to differentiate variceal from non-variceal cause of UGIH, as well as predict large varices, in our study. Ultrasound done in the radiology suite, in CLD patients has been previously used to predict varices [6, 7, 10, 16, 20, 23, 24]. We found that bedside PVFV, done by the emergency physician, in an active UGIH patient was useful in etiology identification in the ED.

Other portal vein indices, such as PVD [7, 8, 15–17, 20, 23] and PCI [7, 20, 23], have also been used in CLD patients without active bleeding as varices predictor. On multivariate logistic regression analysis, we did not find a significant association between these parameters and varices in our patients. However, PCI was found to be a significant parameter for predicting grade of varices. Active hemorrhage, unstable hemodynamics, and ongoing resuscitation may impact the PVD, which needs to be studied further.

Variceal hemorrhage is a well-recognized and frequent complication of chronic liver disease, and our findings reaffirm this, with prior CLD showing a strong association with the presence of varices (Cramer’s *V* = 0.612). Clinically, this supports the principle that any patient with CLD presenting with UGIH should be treated as variceal until proven otherwise, with early initiation of vasoactive agents, antibiotics, and timely endoscopy. However, a substantial proportion of patients present to the ED with UGIH without a prior diagnosis of CLD, and in these individuals, the variceal etiology may not be immediately apparent. In this subgroup as well, spleen diameter, PT, PC/SD ratio and PVFV showed good discriminatory performance, highlighting their value in identifying previously unrecognized portal hypertension.

Among patients with known CLD, while the majority had variceal bleeding, not all UGIH was variceal. In this subgroup, spleen diameter and PVFV remained useful markers for distinguishing variceal from non-variceal sources, underscoring the clinical utility of POCUS-based hemodynamic assessment even when CLD status is already known. These POCUS parameters were particularly useful in patients with Child–Pugh Class A and B.

Fibrosis scores are predictive models developed to assess the likelihood of liver cirrhosis and severe disease based on laboratory markers. Patients with advanced CLD, are likely to have portal hypertension complications. Hence, these scores have been used to predict presence of varices and need for UGI endoscopy in CLD patients [9, 23, 25–28]. We found these scoring systems, namely AAR, APRI, FIB-4, King, and Lok scores, when used in UGIH patients had AUC > 0.7 for predicting varices. Civan et al. used APRI score to predict varices among patients presenting to the emergency department with UGIH. By applying this retrospectively to their cohort, they determined that adherence to established guidelines for administering octreotide and antibiotics would have increased from 56 to 91% if the APRI score had been utilized [5].

FIB-4 is one of the most widely validated non-invasive fibrosis scores and is routinely used to stratify the severity of chronic liver disease, with established thresholds of < 1.3 for ruling out and > 2.67 for ruling in advanced fibrosis [29]. Because it relies solely on laboratory parameters available early in the ED work-up, such as AST, ALT, and platelet count, FIB-4 can be calculated at presentation and may assist clinicians in recognizing previously undiagnosed liver disease. This is clinically important because a significant proportion of patients with variceal hemorrhage present without a documented diagnosis of CLD, and early identification of portal hypertension may influence timely interventions. Several previous studies have evaluated cut-offs for FIB-4 scores in predicting varices and although thresholds vary across cohorts (from 2.8 to 6.4), higher fibrosis scores consistently correlate with advanced portal hypertension and increased likelihood of varices [9, 26, 28]. The cut-offs for the various studied parameters and fibrosis scores are compiled in the supplementary material (Supplementary Tables 7 and 8).

Although fibrosis scores were useful in predicting variceal etiology in our UGIH cohort, the SCoPE score demonstrated superior discriminatory ability, with an AUC of 0.843. This is likely because SCoPE incorporates POCUS-derived spleen diameter and PVFV—parameters that directly reflect the hemodynamic physiology of portal hypertension—while fibrosis scores primarily capture hepatic dysfunction rather than portal flow dynamics.

Our research steps into a newer realm of POCUS by ED physicians and highlights the use of portal vein indices in guided management of UGIH. Additionally, laboratory markers and fibrosis scores could be useful adjuncts. The novel SCoPE score could be an important tool for ED physicians to identify variceal bleeds. Further research would be needed to standardize protocols and establish robust predictive models for variceal etiology of UGIH.

### Limitations

Inter-observer variability is a challenge in POCUS-based assessment, particularly for Doppler measurements such as PVFV. Ultrasound measurements can vary with operator technique, probe positioning, Doppler angle optimization, and overall experience. Reliable estimation of reproducibility ideally requires independent repeat measurements on the same patients by multiple operators, with agreement quantified using statistics such as the Intraclass Correlation Coefficient (ICC) for continuous variables or Cohen’s kappa for categorical parameters. As duplicate blinded assessments were not performed in our study, inter-observer reliability could not be quantified. However, to minimize operator-related variability, all POCUS examinations were performed by the same two investigators, both of whom underwent structured training in portal ultrasound under a radiologist. Prior to patient enrollment, each completed 25 supervised portal vein ultrasound examinations to achieve competence in image acquisition and interpretation. These measures helped standardize technique although formal reproducibility testing remains an important area for future research.

Second, although the operator performing the POCUS examination was blinded to patients’ comorbidities, including prior diagnosis of CLD, physical examination findings such as jaundice, stigmata of chronic liver disease, or visible ascites may inadvertently influence operator perception and could introduce subtle bias during image acquisition or interpretation. We attempted to minimize this effect through blinding to history of comorbidities and structured training for image acquisition. Additionally, obesity and bowel gas may obscure visualization in some patients. The preload on the heart and interplay of other factors, such as increased intra-thoracic or intra-abdominal pressures, may influence portal hemodynamic indices. These factors may cause subtle changes in the portal vein indices during active resuscitation and needs further research.

## Conclusion

Early identification of variceal etiology in UGIH within the ED is critical for guiding optimal treatment strategies. Bedside POCUS parameters, such as spleen diameter and PVFV, along with laboratory markers like platelet count and PT and composite indices such as PC/SD ratio, can help us rapidly identify patients with variceal hemorrhage. Incorporating fibrosis scores into the assessment of patients with active UGIH may further enhance the ability to estimate the likelihood of varices.

The novel **SCoPE score**, which is a composite score that includes spleen diameter, PVFV and PT, can be a valuable ED-based tool for rapid identification of variceal bleed in UGIH patients, particularly in resource-limited settings where immediate endoscopy may not be available.

### Future directions

Prospective studies integrating POCUS into clinical decision-making pathways for patients with active UGIH in the ED may help us study its impact on patient outcomes. Predictive models using combination of these parameters maybe useful to establish protocols in ED. Artificial intelligence and machine learning of portal vein indices POCUS could improve diagnostic capabilities by automating image analysis and minimizing reliance on the operator.

## Supplementary Information

Below is the link to the electronic supplementary material.

Supplementary file1 (DOCX 395 KB)

## Data Availability

Data supporting the findings of this study are available from the corresponding author on reasonable request.

## Abbreviations

UGIH: Upper gastrointestinal hemorrhage
UGIE: Upper gastrointestinal endoscopy
CLD: Chronic liver disease
ED: Emergency Department
POCUS: Point of care ultrasound
PVFV: Portal vein flow velocity
PC/SD: Platelet count/spleen diameter ratio
PVD: Portal vein diameter
PCI: Portal congestion index
Portal CSA: Portal vein cross-sectional area
ROC: Receiver operating curve
AUC: Area under the curve
