# Supplementary material for: A portal into hemodynamics: utility of portal vein ultrasound in predicting variceal etiology of upper gastrointestinal hemorrhage in the emergency department
Source: Intern Emerg Med. 2025 Dec 23;21(8):3085–98. doi: 10.1007/s11739-025-04229-2 (PMC13751866; doi:10.1007/s11739-025-04229-2)
Supplement: Supplementary file 1 [file 11739_2025_4229_MOESM1_ESM.docx]

**SUPPLEMENTARY MATERIAL**

**Supplementary Figure 1**: Flowchart demonstrating patients presenting with UGIH to the ED enrolled into the study

**Supplementary Table 1:** Diagnostic accuracy of SCoPE score and Fibrosis scores for predicting varices in UGIH

| Variables | AUC | OR  (95% C.I) | Cut-offs | Sensitivity (%) | Specificity (%) | PPV  (%) | NPV  (%) | Diagnostic accuracy (%) | LR  (+) | LR  (-) |
| --- | --- | --- | --- | --- | --- | --- | --- | --- | --- | --- |
| SCoPE | 0.843* | 1.633  (1.417-1.883) | **>/=3** | 79.2 | 77 | 83.2 | 71.8 | 78.2 | 3.4 | 0.27 |
| AAR | 0.663* | 1.400  (1.079-1.816) | **>1.94** | 61.3 | 68 | 73 | 55.4 | 64.1 | 1.92 | 0.57 |
| APRI | 0.742* | 1.253  (1.051-1.494) | **>0.86** | 75 | 65.3 | 75.2 | 65 | 71 | 2.15 | 0.39 |
| Fib-4 | 0.699* | 1.143  (1.054-1.239) | **>3.59** | 68 | 68 | 75 | 60 | 68 | 2.12 | 0.472 |
| King | 0.738* | 1.006  (1.001-1.011) | **>20.34** | 79.2 | 63.5 | 75.7 | 68.1 | 72.8 | 2.17 | 0.327 |
| Lok | 0.775* | 103.64  (23.29-461.06) | **>0.65** | 91.5 | 67 | 74 | 81.5 | 76.1 | 1.99 | 0.16 |
| *P<0.005 | | | | | | | | | |  |

**Supplementary Table 2:** Comparison of POCUS and laboratory parameters between Patients with Varices and Patients without Varices on UGIE in all patients with CLD (n=122)

| Parameter | Varices absent (n=17) | Varices present (n=105) | p-value |
| --- | --- | --- | --- |
| Portal vein diameter (mm) | 9.59 ± 1.95 | 9.86 ± 2.72 | 0.693* |
| **Spleen diameter (cm)** | **10.73 ± 2.03** | **12.46 ± 2.30** | **0.004^*^** |
| **Portal vein velocity (cm/s)** | **14.20 (11.75-17.65)** | **11.5 (8.90- 14)** | **0.035^#^** |
| Portal vein CSA (cm^2^) | 0.79 (0.58-0.94) | 0.74 (0.52-1.09) | 0.897^#^ |
| Portal vein congestion index | 0.05 (0.04-0.08) | 0.06 (0.04-0.09) | 0.223^#^ |
| Platelet (cu mm)/  Spleen diameter(mm) | 1128 (851-1744) | 1050 (726-1537) | 0.174^#^ |
| Ascites present | 5 (29.4%) | 47 (44.8%) | 0.235^^^ |
| Blood Flow pattern  Hepatopetal-1  Hepatofugal-2  Mixed-3 | 76.5%  11.8%  11.8% | 56.2%  21.9%  21.9% | 0.288^^^ |
| Hematocrit | 25.0 (15.5- 31.00) | 26 (20-30.5) | 0.597^#^ |
| WBC | 10.2 (7.05-14.85) | 10 (7.1-13.95) | 0.994^#^ |
| Platelets | 129 (106.5 -193) | 134 (86-186) | 0.478^#^ |
| Creatinine | 1.41 (0.77-1.70) | 0.98 (0.72-1.36) | 0.175^#^ |
| Urea | 55 (30.5-122.5) | 41.00 (31-64.50) | 0.132^#^ |
| Total bilirubin | 1.54 (0.9-3.4) | 2.08 (1.08-5.63) | 0.292^#^ |
| Direct bilirubin | 0.78 (0.54-2.53) | 1.2 (0.55-2.46) | 0.688^#^ |
| AST | 60 (36-111.5) | 72 (49.5-116.50) | 0.387^#^ |
| ALT | 36 (18.85-51.5) | 30 (21.65-50.9) | 0.900^#^ |
| ALP | 87 (64-133) | 102 (82.50-132.5) | 0.146^#^ |
| Albumin | 2.81± 0.82 | 2.84±0.58 | 0.901* |
| PT | 16.64±5.14 | 16.81±5.18 | 0.900* |
| aPTT | 30.95±10.81 | 30.79±8.51 | 0.945* |
| *Independent Student t-test, #Mann-Whitney U test, ^Chi-Square test | | | |

**Supplementary Figure 2**

2 (a): ROC curve analysis of spleen diameter for predicting the presence of varices in UGIH in all CLD patients (AUC=0.713)

2 (b) ROC curve analysis of PVFV for predicting the absence of varices in UGIH in all CLD patients (AUC=0.66)

2 (a)	2 (b)

**Supplementary Table 3**: Child–Pugh–wise comparison of spleen diameter and PVFV between patients with and without varices on UGIE among those with CLD (n = 122)

|  | No varices (n= 17) | **Varices (n= 105)** | P value |
| --- | --- | --- | --- |
| **PVFV (cm/s)** | | | |
| Child Pugh A (n=23) | 14.20 (12-16.7) | 10.45 (8.94-11.95) | **0.015^#^** |
| Child Pugh B (n=40) | 17.65 (15.57-23.10) | 11.25 (9.65-13.55) | **0.002^#^** |
| Child Pugh C (n=59) | 10.5 (7.2-15.8) | 12 (8.15-16.15) | 0.651**^#^** |
| **Spleen diameter (cm)** | | | |
| Child Pugh A (n=23) | 9.79 ± 1.51 | 11.99 ± 2.77 | **0.024*** |
| Child Pugh B (n=40) | 11.16 ± 2.04 | 12.01 ± 2.53 | 0.524* |
| Child Pugh C(n=59) | 11.53 ± 2.43 | 12.91 ± 1.92 | 0.109* |
| *Independent Student t-test, #Mann-Whitney U test | | | |

**Supplementary Figure 3:**

3 (a): ROC curve analysis of spleen diameter for predicting the presence of varices in UGIH among Child–Pugh A CLD patients(AUC=0.768)

3(b)	3(c)

3 (b) ROC curve analysis of PVFV for predicting the absence of varices in UGIH among Child Pugh A CLD patients (AUC=0.817)

3 (c) ROC curve analysis of PVFV for predicting the absence of varices in UGIH among Child Pugh B CLD patients (AUC=0.934)

**Supplementary table 4:**

Comparison of POCUS parameters between Patients with Varices and Patients without Varices on UGIE in newly diagnosed CLD (n=32)

| Parameter | Varices absent (n=7) | Varices present (n=25) | p-value |
| --- | --- | --- | --- |
| Portal vein diameter (mm) | 9.08 ± 1.63 | 9.74 ± 2.93 | 0.579* |
| Spleen diameter (cm) | 10.33 ± 2.00 | 12.02 ± 1.98 | 0.058^*^ |
| Portal vein velocity (cm/s) | 15 (12.70-18.00) | 9.8 (7.20- 11.65) | **0.001^#^** |
| Portal vein CSA (cm^2^) | 0.68 (0.57-0.85) | 0.80 (0.51-1.10) | 0.721^#^ |
| Portal vein congestion index | 0.045 (0.035-0.052) | 0.073 (0.05-0.119) | **0.018^#^** |
| Platelet (cu mm)/Spleen diameter(mm) | 1460 (9682-1723) | 1030 (796-1632) | 0.261^#^ |
| Ascites present | 1 (14.3%) | 11 (44%) | 0.151^^^ |
| Blood Flow pattern  Hepatopetal-1  Hepatofugal-2  Mixed-3 | 85.7%  14.3%  0% | 48%  24%  28% | 0.167^^^ |
| *Independent Student t-test, #Mann-Whitney U test, ^Chi-Square test | | | |

**Supplementary table 5:**

Comparison of POCUS and laboratory parameters between Patients with Varices and Patients without Varices on UGIE among study participants without prior diagnosis of CLD (n=91)

| Parameter | Varices absent (n=65) | Varices present (n=26) | p-value |
| --- | --- | --- | --- |
| Portal vein diameter (mm) | 8.88 ± 2.67 | 9.78 ± 2.88 | 0.163* |
| Spleen diameter (cm) | 9.56 ± 2.09 | 12.08 ± 1.97 | **<0.001^*^** |
| Portal vein velocity (cm/s) | 15.00 (11.25-19.00) | 9.80 (7.30-11.85) | **<0.001^#^** |
| Portal vein CSA (cm^2^) | 0.63 (0.50-0.80) | 0.80 (0.51-1.08) | 0.076^#^ |
| Portal vein congestion index | 0.04 (0.03-0.05) | 0.07 (0.05-0.11) | **<0.001^#^** |
| Platelet (cu mm)/Spleen diameter(mm) | 2195 (1333-3236) | 1040 (800-1669) | **<0.001^#^** |
| Ascites present | 6 (9.2%) | 11 (42.3%) | **<0.001^^^** |
| Blood Flow pattern  Hepatopetal-1  Hepatofugal-2  Mixed-3 | 61.5%  9.2%  29.2% | 46.2%  23.1%  30.8% | 0.175^^^ |
| Hematocrit | 29.0 (20.20- 36.00) | 26 (21-32.5) | 0.266^#^ |
| WBC | 10.2 (6.95-14.30) | 10.15 (8.05-14.20) | 0.765^#^ |
| Platelets | 210 (142 -260.5) | 130.5 (98.75-193.5) | **<0.001^#^** |
| Creatinine | 1.03 (0.8-1.43) | 0.93 (0.71-1.24) | 0.232^#^ |
| Urea | 47 (24-77.5) | 38.00 (31.50-64.00) | 0.527^#^ |
| Total bilirubin | 0.68 (0.35-1.15) | 1.72 (0.72-3.22) | **<0.001^#^** |
| Direct bilirubin | 0.31 (0.14-0.55) | 0.61 (0.35-1.41) | **<0.001^#^** |
| AST | 35 (20.5-70) | 70 (40.25-101.50) | **0.002^#^** |
| ALT | 21 (14.15-38) | 27.5 (23.60-55.45) | **0.010^#^** |
| ALP | 72 (57.5-98) | 96.0 (76.50-117.0) | **0.005^#^** |
| Albumin | 3.41±0.74 | 3.06±0.56 | **0.038*** |
| PT | 12.41±2.62 | 15.42±3.97 | **<0.001*** |
| aPTT | 26.67±10.33 | 28.21±5.47 | 0.498* |
| *Independent Student t-test, #Mann-Whitney U test, ^Chi-Square test | | | |

**Supplementary Figure 4:**

4 (a)	4 (b)

4 (a): ROC curve analysis of spleen diameter (AUC= 0.819) and PCI (AUC=0.755) for predicting the presence of varices in UGIH among patients without prior history of CLD

4 (b): ROC curve analysis of PVFV (AUC= 0.773) and PC/SD (AUC=0.801) for predicting the absence of varices in UGIH among patients without prior history of CLD

4 (c)	4 (d)

4 (c): ROC curve analysis of ALP (AUC=0.678 ), ALT (AUC=0.673), AST (AUC= 0.698), PT (AUC=0.814) and total bilirubin (AUC= 0.737) for predicting the presence of varices in UGIH among patients without prior history of CLD

4 (d): ROC curve analysis of albumin (AUC= 0.647) and Platelets (AUC=0.745) for predicting the absence of varices in UGIH among patients without prior history of CLD

**Supplementary table 6:**

Comparison of POCUS parameters between Patients with Varices and Patients without Varices on UGIE among study participants not on non-selective beta blockers (n=129)

**Supplementary figure 5:**

| Parameter | Varices absent (n=69) | Varices present (n=60) | p-value |
| --- | --- | --- | --- |
| Portal vein diameter (mm) | 9.02 ± 2.70 | 9.94 ± 2.79 | 0.058* |
| Portal vein velocity (cm/s) | 14 (10.55- 18.45) | 10.7 (8.54- 13.95) | **<0.001^#^** |
| Portal vein CSA (cm^2^) | 0.64 (0.50-0.83) | 0.75 (0.53-0.95) | 0.074^#^ |
| Portal vein congestion index | 0.04 (0.03-0.06) | 0.07 (0.04-0.09) | **<0.001^#^** |

5 (a)	5 (b)

5 (a): ROC curve analysis of PVFV (AUC= **0.676**) for predicting the absence of varices in UGIH among patients not on non-selective beta blockers

5 (b): ROC curve analysis of PCI (AUC=0.693) for predicting the presence of varices in UGIH among patients not on non-selective beta blockers

**Supplementary Figure 6**: Correlation of systolic blood pressure with portal vein flow velocity (6 a) and portal vein diameter (6 b)

6(a)

6 (b)

**Supplementary table 7:** Summary of cut-off values for POCUS parameters from previous studies predicting varices

| Studies | Place of Study | Study Population | PVD (mm) | PVFV (cm/s) | PCI | SD (cm) | PC (per mm3)/SD  (mm) | Platelet counts (10^3^/µL) |
| --- | --- | --- | --- | --- | --- | --- | --- | --- |
| Naik, et al | **Karnataka, India** | **UGIH** |  | **< 14** |  | **≥ 12** | **< 1741** | **< 155** |
| Nouh et al (20) | Egypt | CLD | 10.4 | 15.2 | 0.1 |  | 604 |  |
| Bhattarai et al (17) | Nepal | CLD | 12.25 |  |  | 13.9 |  |  |
| Elkenawy et al (6) | Egypt | CLD |  | 19 |  |  |  | 1,40,000/µL |
| Katwal et al (7) | Nepal | CLD | 13 | 19 | 0.1 |  |  |  |
| Shanker et al (15) | Jaipur, India | CLD | 12.2 |  |  | 13.5 |  |  |
| Wasif Khan et al (10) | Lahore | CLD |  | 18 |  |  |  |  |
| Zhang et al (16) | China | CLD | 14.3 | 19.6 |  | 16.2 |  |  |
| Shastri et al (23) | Vadodara, India | CLD | 13 | 16 | 0.1 |  |  |  |
| Zironi et al (24) | Italy | CLD |  | 15 |  |  |  | 40,000/µL |
| Cherian et al (8) | Chennai, India | CLD | 13 |  |  | 15 | 666 |  |
| Patil et al (21) | Bhubaneshwar, India | CLD |  |  |  |  | 1400 |  |
| Baig et al (22) | Karnataka, India | CLD |  |  |  |  | 1014 |  |

| Studies | Study Population | APRI | AAR | FIB-4 | King | Lok |
| --- | --- | --- | --- | --- | --- | --- |
| **Naik, et al** | **UGIH** | **0.86** | **1.94** | **3.59** | **20.34** | **0.65** |
| Pandey et al (9) | CLD | 1.4 | 1.7 | 6.4 | 46.5 | 0.97 |
| Civan et al (5) | UGIH | 1.4 |  |  |  |  |
| Liu et al (26) | CLD | 1.33 |  | 3.83 | 19.38 | 1.62 |
| Zhang et al (25) | CLD | 1.4 | 1.3 |  |  |  |
| Tarar et al (27) | CLD |  |  |  |  | 0.335 |
| Hassan et al (28) | HCV- related CLD | 0.85 | 0.67 | 2.8 | 24.7 |  |

**Supplementary Table 8 :** Summary of Fibrosis Score Cut-off Values from Previous Studies for Variceal Prediction
